# Supplementary figures and images for: Structure and Properties of High-Entropy Boride Ceramics Synthesized by Mechanical Alloying and Spark Plasma Sintering
Source: Materials (Basel). 2023 Oct 18;16(20):6744. doi: 10.3390/ma16206744 (PMC10608203; doi:10.3390/ma16206744)

Figure S1. MA 7.5h  $(\text{TiTaNb})_{0.3}\text{Hf}_{0.35}\text{Zr}_{0.35}\text{B}_2$ .

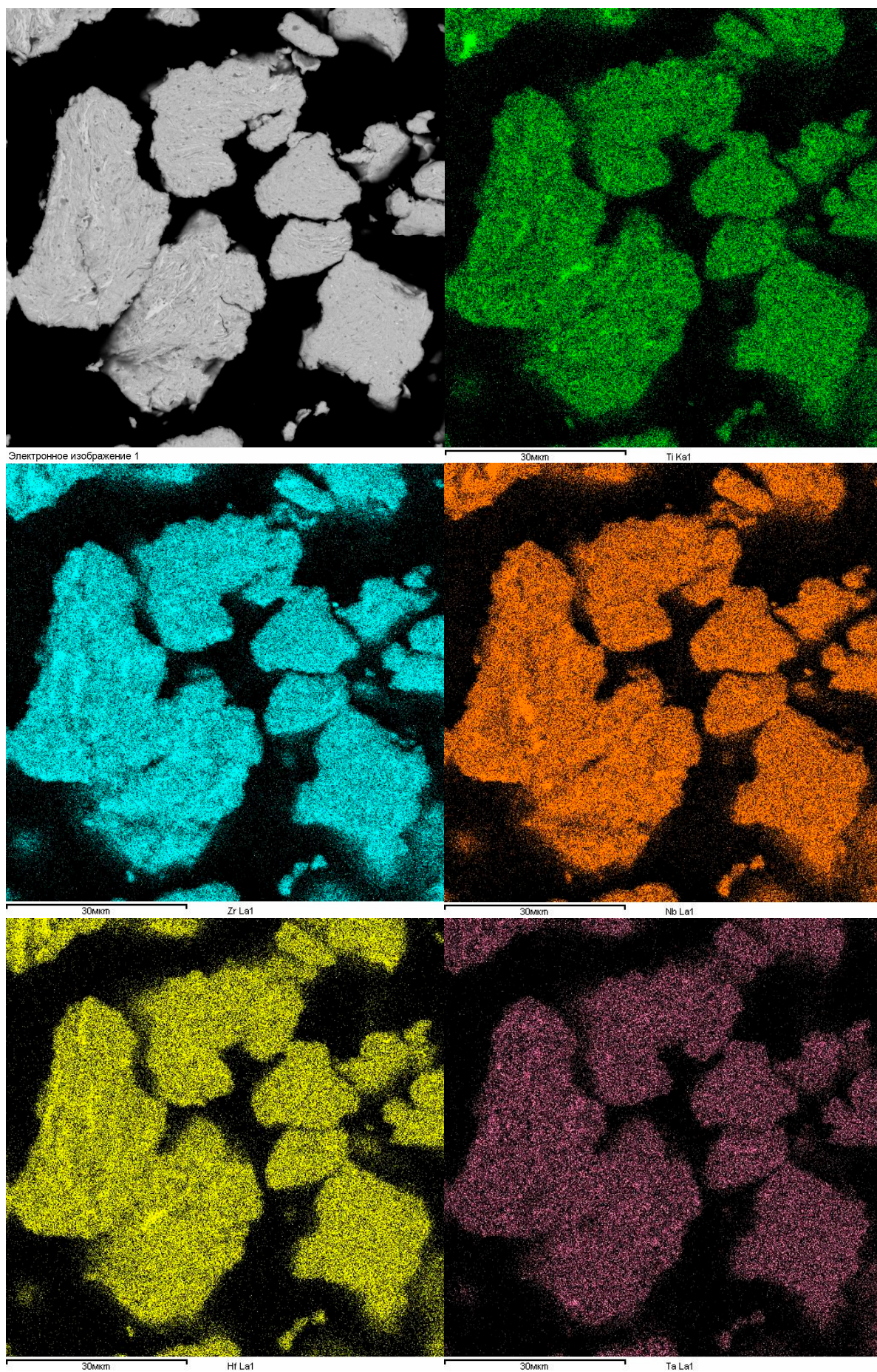

Supplement: Supplementary file 1 [file materials-16-06744-s001.zip › materials-2644598-supplementary.pdf]
